# Heterogeneity in risk of prostate cancer: A Swedish population‐based cohort study of competing risks and Type 2 diabetes mellitus

**DOI:** 10.1002/ijc.31587

**Published:** 2018-08-10

**Authors:** Christel Häggström, Mieke Van Hemelrijck, Hans Garmo, David Robinson, Pär Stattin, Mark Rowley, Anthony C.C. Coolen, Lars Holmberg

**Affiliations:** ^1^ Department of Surgical Sciences Uppsala University Uppsala Sweden; ^2^ Department of Biobank Research Umeå University Umeå Sweden; ^3^ King's College London, School of Cancer and Pharmaceutical Sciences Translational Oncology & Urology Research (TOUR) London United Kingdom; ^4^ Institute of Environmental Medicine Karolinska Institute Stockholm Sweden; ^5^ Regional Cancer Centre Uppsala/Örebro Uppsala Sweden; ^6^ Department of Urology Ryhov Hospital Jönköping Sweden; ^7^ Institute for Mathematical and Molecular Biomedicine King's College London London United Kingdom; ^8^ Saddle Point Science London United Kingdom

**Keywords:** survival analysis, competing risks, latent class, prostate cancer, Type 2 diabetes mellitus

## Abstract

Most previous studies of prostate cancer have not taken into account that men in the studied populations are also at risk of competing event, and that these men may have different susceptibility to prostate cancer risk. The aim of our study was to investigate heterogeneity in risk of prostate cancer, using a recently developed latent class regression method for competing risks. We further aimed to elucidate the association between Type 2 diabetes mellitus (T2DM) and prostate cancer risk, and to compare the results with conventional methods for survival analysis. We analysed the risk of prostate cancer in 126,482 men from the comparison cohort of the Prostate Cancer Data base Sweden (PCBaSe) 3.0. During a mean follow‐up of 6 years 6,036 men were diagnosed with prostate cancer and 22,393 men died. We detected heterogeneity in risk of prostate cancer with two distinct latent classes in the study population. The smaller class included 9% of the study population in which men had a higher risk of prostate cancer and the risk was stronger associated with class membership than any of the covariates included in the study. Moreover, we found no association between T2DM and risk of prostate cancer after removal of the effect of informative censoring due to competing risks. The recently developed latent class for competing risks method could be used to provide new insights in precision medicine with the target to classify individuals regarding different susceptibility to a particular disease, reaction to a risk factor or response to treatment.

## Abstract 2

**What's new?**

Men with type 2 diabetes have a decreased risk of developing prostate cancer but metabolic disease is associated with a shorter life expectancy. Here the authors applied a new method to handle competing risks and cohort heterogeneity and found no association between type 2 diabetes mellitus and prostate cancer risk after isolating the effect from competing risks. This method may support precision medicine by correctly classifying individuals regarding susceptibility to a particular cancer or response to treatment.

## Introduction

Underlying assumptions in most common methods for survival analysis are that censoring is non‐informative and that the association between risk factors and the event of interest is homogenous in the population studied.^1^ However, these assumptions are often violated or neglected. First, informative censoring may happen when the studied risk factor (e.g., smoking, obesity, metabolic syndrome) is associated with death and a shorter life‐expectancy.^2^, ^3^, ^4^ Methods based on competing risk analysis can provide reliable real‐life estimates of risk in these study settings, but results are often difficult to interpret in terms of etiological hypotheses,^5^, ^6^ especially if the risk factor is associated with both time to death and the risk of the disease under study. Secondly, risk factors or other studied covariates (e.g. treatments, drugs, and dietary exposures) may have different associations with the risk within sub classes of the population studied,^7^, ^8^ and previous reports of heterogeneity in cancer risk suggested that the majority of the cases are arising from a minor susceptible part of the population.^9^ Thus, there may be latent classes within a population with a known or unknown geno‐ or phenotype that render them to higher susceptibility of disease, or high risk in association with a specific covariate, or positive or negative response to a specific treatment, in line with the theories behind precision medicine.^10^

Researchers investigating metabolic aberrations, metabolic diseases, or drugs for metabolic diseases, and risk of prostate cancer are facing a complex task. Observational studies suggest that men with Type 2 diabetes mellitus (T2DM),^11^, ^12^, ^13^, ^14^, ^15^, ^16^, ^17^ and men on anti‐diabetic drugs,^18^, ^19^, ^20^ have a decreased risk of developing prostate cancer. Moreover, other studies indicate that men with metabolic aberrations have a higher risk of aggressive or fatal prostate cancer.^21^, ^22^, ^23^, ^24^ Due to the higher age when prostate cancer generally occurs, competing risk in terms of death is an issue,^25^, ^26^ especially since metabolic disease is associated with a shorter life‐expectancy. One previous study concluded that individuals with diabetes at a given age have a smaller lifetime risk of cancer than individuals without diabetes at the same age, attributable to the higher mortality rates among individuals with diabetes.^27^ Moreover, they noted that differences in cancer occurrence between individuals with and without diabetes were a quantitatively smaller problem than the differences in mortality between the two groups.^27^ In line with these results, we have previously investigated prostate cancer risk in relation to other competing causes of death and reported that the decreased risk of prostate cancer among men with metabolic aberrations is of much smaller quantity than the increased risk of death among those men, as compared to men with normal metabolic levels. 28 We have also used other methods for competing risks to investigate similar research questions with respect to prostate cancer,^29^, ^30^ but to the best of our knowledge there is no established method that yields straight forward interpretations to analyze etiological associations within these study settings.

Based on these limitations in conventional survival analysis some of the present authors recently developed a method to handle competing risks and cohort heterogeneity based on latent class analysis.^31^ The aim of the current study was to investigate heterogeneity in risk of prostate cancer classified into risk categories defined by tumor characteristics at diagnosis by applying this method. We further aimed to elucidate the etiological association between T2DM and risk of prostate cancer, and to compare the results with results derived from conventional methods for survival analysis.

## Material and Methods

### Participants

We applied a method based on latent class analysis for competing risks,^31^ on a prospective cohort study in the comparison cohort of Prostate Cancer Data base Sweden (PCBaSe) 3.0. For each man with prostate cancer in the National Prostate Cancer Register of Sweden, five prostate cancer‐free men were randomly selected from the background population into the comparison cohort, matched on birth year and county of residence.^32^ By using the Swedish 10‐digit personal identity number, the men were linked to a number of national health care registers and demographic databases. The study was approved by the Research Ethics Board at Umeå University, Sweden.

For the current study, we selected men who entered the comparison cohort between January 1, 2007, and December 31, 2009, and were between 55 and 84 years old. The men in the study were followed from entry date until date of prostate cancer diagnosis, date of death, or December 31 2014, whichever occurred first. Time in follow‐up was used as a timescale in all analyses.

### Covariates in study

We retrieved data from the National Patient Register on discharge diagnoses from hospital admissions up to ten years prior to the date of inclusion in the comparison cohort. These data were used to calculate the Charlson Comorbidity Index (CCI), categorised into no comorbidity (0), mild (1), moderate (2), and severe (3 or more) comorbidity as described previously.^33^, ^34^ Data on educational level were retrieved from the Longitudinal Integration Database for Health Insurance and Labor Market Studies at Statistics Sweden, and were categorized into three groups based on duration of education: ≤9 years, 10–12 years, and ≥13 years, which corresponded to elementary school, secondary school, and higher education.^35^ Status of T2DM was defined by anti‐diabetic drug prescriptions classified according to the Anatomical Therapeutic Chemical Classification System from the Prescribed Drug Register. We retrieved data of metformin (A10BA or A10BD), insulin (A10A) and sulphonylurea (A10BB), and classified T2DM status as users of no anti‐diabetic drugs, metformin, or insulin/sulphonylurea. This categorization were based on the national guidelines of diabetes care and pattern of use of anti‐diabetic drugs in Sweden.^36^ We used age at start of study, T2DM status, education level and CCI as covariates in all analyses. T2DM status and CCI were recorded at start of study. Covariates were transformed to *z*‐scores (mean = 0, standard deviation = 1) prior to analysis.

### Endpoints assessed

Data on prostate cancer diagnosis was obtained from the National Prostate Cancer Register of Sweden which includes information on date of diagnosis, age at diagnosis, tumour stage and differentiation, serum levels of prostate‐specific antigen (PSA) at time of diagnosis, and primary treatment.^31^ Prostate cancer risk categories were defined at diagnosis according to a modification of the National Comprehensive Cancer Network Guideline:^37^ Low‐risk: T1‐2, Gleason score of 2–6 and PSA < 10 ng/ml; intermediate‐risk: T1‐2, Gleason score 7 and/or PSA 10–20 ng/ml; high‐risk: T3 and/or Gleason score 8–10 and/or PSA 20–50 ng/ml; metastatic disease: T4 and/or N1 and/or PSA 50–100 ng/ml (regional metastases) or M1 and/or PSA > 100 ng/ml (distant metastases).^32^ We clustered these risk categories into favorable‐risk prostate cancer (low‐ and intermediate‐risk), and aggressive prostate cancer (high‐risk and metastatic disease).^38^ Date and cause of death were obtained from the Cause of Death Register, and deaths from cardiovascular diseases were defined as codes I00.0‐I99.9 (International Classification of Diseases, 10th revision). We used four endpoints in the analyses: favorable‐risk and aggressive prostate cancer, death of cardiovascular diseases and death of other causes.

### Survival analysis

We investigated heterogeneity in risk of prostate cancer, and elucidated the association between T2DM status and risk of prostate cancer to differentiate the effect of informative censoring from competing endpoints, with the latent class model for competing risks described previously,^31^ and also briefly described below. In comparison to conventional survival models, the model does not assume homogenous associations between covariates and endpoints and takes into account simultaneous risks of all four endpoints. It assumes that the study population may be comprised of one or several latent classes, and that risk differences between classes are induced by latent heterogeneity, that is heterogeneity not captured by covariates, and can be quantified by relative frailty and/or variability in associations of base hazard rates. The assumption of proportional hazards is only made for each endpoint and each latent class separately, but is not required to be valid for the full study population collectively. If no latent classes are found in the study population, the method will automatically reduce to a model with similar settings as the Cox proportional hazards model.

Associations with covariates and latent class membership on risk of all endpoints were investigated by calculations of hazard ratios (HR) from the latent class model for competing risk analysis. The HR for latent class membership are defined by relative frailties. The calculations were performed with the software package Advanced Latent Class Prediction and Competing Risk Analysis version 0.2, (ALPACA, A.C.C. Coolen, M. Rowley, M. Inoue, London, UK). The results from the latent class model for competing risk analysis were compared to HR from Cox and Fine and Gray regression models.^39^ In the Cox models, we calculated the risk of each endpoint separately and censored for all other endpoints, while in Fine and Gray regression models, all other endpoints were handled as competing risks. These models were calculated by STATA MP/2 version 14 (StataCorp LP, College Station, Texas).

### Latent class model for competing risk

As described in detail previously,^31^ latent class analysis in ALPACA is performed in two stages: during the *parameter estimation stage*, model estimates are obtained for many candidate models, covering a range of latent classes, base hazard rate complexities, and degrees of permitted heterogeneity. Due to its stochastic nature, this process is performed multiple times until consistent estimates are obtained. At the *model selection stage* the relative probabilities of all candidate models are computed and the model with the greatest probability of describing the study data, the optimal model, is determined. Bayesian model selection balances the need for model complexity with the evidence available in the data to support such complexity. The base hazard rate parametrisation is based on a spline construction, where the inferred number of spline points increases with the complexity of the base hazard rate. In our study, we included latent class models with one to four latent classes, and with up to ten spline time points.

Based on the optimal model, we analysed the base hazard rate, covariate HRs, latent class membership HRs (relative frailty), and survival functions, both under the influence of competing risks (crude) and isolated from the effect of censoring due to competing risks (marginal), separately for each latent class. We here quantified the association of the covariates on the endpoints by calculating covariate HRs and quantified the association attributed by the class membership on the endpoints by calculating class membership HRs, that is the association of relative frailty due to factors not accounted for in our study. Characteristics of study participants were analysed in relation to the most probable latent class they were assigned to. In order to assess the effect of censoring due to competing risks we graphically compared the crude and marginal survival functions.

## Results

Mean age at start of study was 70 years (SD = 7 years). During a mean follow‐up of 6 years (SD = 2), 3,397 men were diagnosed with favorable‐risk prostate cancer, 2639 men with aggressive prostate cancer, 9,165 died of cardiovascular diseases and 13,228 died of other causes without no previously diagnosis of prostate cancer. Baseline characteristics of the full study population are shown in Table 1.

**Table 1:** Baseline characteristics of the study population of 126,482 men

		*N*	%
**Age at start of study**	55–59 years	12,511	10
	60–64 years	26,095	21
	65–69 years	29,774	24
	70–74 years	24,450	19
	75–79 years	19,653	16
	80–84 years	13,999	11
**Year of selection into comparison cohort**	2007	39,957	32
	2008	39,355	31
	2009	47,170	37
**Educational level** a	Low^b^	52,496	42
	Intermediate	47,147	37
	High	26,839	21
**Charlson Comorbity Index**	No comorbidity (0)	93,671	74
	Mild comorbidity (1)	16,834	13
	Moderate comorbidity (2)	9,313	7
	Severe comorbidity (3 or more)	6,664	5
**Type 2 diabetes mellitus status**	No anti‐diabetic drugs	111,900	88
	Metformin	4,552	4
	Insulin/sulphonylurea^c^	10,030	8

a Educational level categorised as low (≤9 years of school), intermediate (10–12 years), and high (≥13 years), corresponding to mandatory school, high school, and college or university.

b Educational level missing for 2006 men (2%); these men were included in the group with low educational level.

c Ordered variable such as men in the insulin/sulphonylurea group could also use metformin.

Based on the latent class model for competing risks, the study population consists with high probability of two distinct latent classes. Class 1 included 115,623 (91%) men in the study, while Class 2 included 10,859 (9%) men. Men in Class 1 were typically younger and with fewer comorbidities than men in Class 2, (Supporting Information Table 1). At the end of follow‐up, 85% of the men in Class 1 were alive and free of prostate cancer, 6% had died of cardiovascular diseases and 9% from other causes, while in Class 2 all men were either dead, 23% from cardiovascular diseases and 24% from other causes, or had been diagnosed with prostate cancer (31% with favorable‐risk and 22% with aggressive prostate cancer; Table 2).

**Table 2:** Endpoint distribution and frailty differences in the two latent classes

	Class 1 (91%, *N* = 115,623)		Class 2 (9%, *N* = 10,859)			Class membership^b^
Status at end of study	*N*	%	*N*	%	Relative frailty^a^	HR (95% CI)
**Alive and free of prostate cancer**	98,053	85	0	0	NA	NA
**Diagnosis of favorable‐risk prostate cancer**	68	0	3,329	31	2.8	16.4 (7.1, 38.3)
**Diagnosis of aggressive prostate cancer**	211	0	2,428	22	2.1	8.1 (4.5, 14.7)
**Death of cardiovascular diseases**	6,642	6	2,523	23	0.1	1.1 (1.6, 1.7)
**Death of other causes**	10,649	9	2,579	24	0.5	1.6 (1.2, 2.2)

a Relative contribution due to class membership defined as relative frailty factor = abs[frailty (Class 1)]‐abs[frailty (Class 2)].

b Association due to class membership: HR = exp(relative frailty factor), 95% confidence intervals were calculated via *z*‐scores from the averages and standard deviations of the regression parameters.

Men in Class 2 were frailer than men in Class 1 with respect to the risk of all four endpoints. The relative frailty between the classes was largest for favorable‐risk and aggressive prostate cancer, with HRs for class membership of 16.4, 95% confidence interval: 7.1, 38.3, for favorable‐risk prostate cancer and 8.1, 95% confidence interval: 4.5, 14.7, for aggressive prostate cancer. All covariate HRs were weaker than the class membership HRs on risk of favorable‐risk and aggressive prostate cancer, (Table 3).

**Table 3:** Statistical significant class‐specific hazard ratios (HRs) for covariates and endpoints in the two latent classes. All covariates transformed to z‐scores (mean = 0, SD = 1) and endpoints are included in the models and HRs are calculated per unit increase

Covariate	Diagnosis of favorable‐risk prostate cancer	Diagnosis of aggressive prostate cancer	Death of cardiovascular diseases	Death of other causes
**Class 1**	**Endpoint**			
**Age**	0.63 (0.46–0.85)	2.01 (1.27–3.19)	3.09 (2.73–3.50)	6.12 (5.11–7.34)
**Type 2 diabetes mellitus status** a	–	–	1.16 (1.08–1.24)	1.25 (1.86–1.32)
**Education level** b	–	–	0.68 (0.63–0.75)	0.79 (1.74–0.86)
**Charlson Comorbidity Index** c	–	–	2.85 (2.56–3.17)	1.26 (1.15–1.38)
**Class 2**	**Endpoint**			
**Age**	0.88 (0.79–0.98)	3.31 (2.76–3.97)	26.12 (11.55–59.10)	1.54 (1.36–1.87)
**Type 2 diabetes mellitus status** a	–	–	–	0.78 (0.69–0.89)
**Education level** b	1.33 (1.16–1.53)	–	0.78 (0.67–0.93)	0.76 (0.64–0.90)
**Charlson Comorbidity Index** c	0.73 (0.58–0.92)	0.68 (0.50–0.92)	2.20 (1.83–2.66)	7.57 (5.45–10.52)

a Defined as 0 = no anti‐diabetic drugs, 1 = metformin, 2 = insulin or sulphonylurea.

b Defined as 1 = low, 2 = intermediate, 3 = high educational level.

c Defined as 0 = no comorbidities, 1, 2, 3 or more comorbidities.

**Figure 1:**
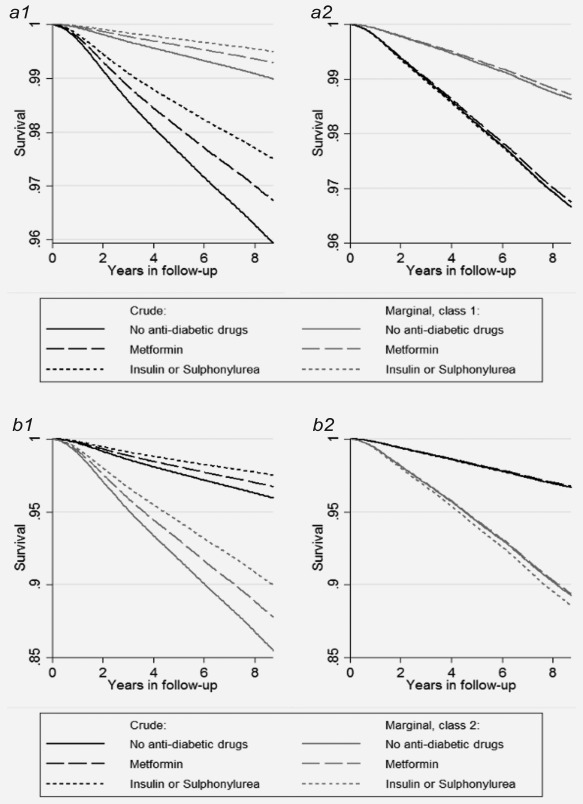
Marginal and crude survival curves separately for Type 2 diabetes mellitus (T2DM) status (*a*) in Class 1 and (*b*) Class 2 on risk of (1) favorable‐risk prostate cancer and (2) aggressive prostate cancer. Black lines are the crude survival curves under influence of competing risks, gray lines are the marginal survival curves isolated from the effect of censoring from competing risks. If the crude and the marginal survival curves are equal there are no effects of competing risks. If the crude survival curve shows worse survival than the marginal survival curve, as in a1 and a2, there is an overestimation of the risk (false aetiology). If the crude survival curve shows better survival than the marginal survival curve, as in b1 and b2, there is an underestimation of the risk (false protectivity). The crude survival curves are not class‐specific.

We found no associations between T2DM status and risk of favorable‐risk and aggressive prostate cancer in any of the classes, but an association with death of cardiovascular diseases and other causes in Class 1, and in Class 2 an inverse association with death of other causes. By visually observing the crude and marginal survival functions for T2DM status, we found that informative censoring due to competing risks had opposite effects on the survival curves in the both classes (Figure 1). The scales were different between the figures and the differences between the crude and marginal survival curves were far higher in Class 2. In that class, the crude survival curve (under influence of competing risks) underestimated the risk as compared to the marginal survival curve (isolated from the effect of competing risks), while in Class 1 we found a smaller, but opposite effect.

In the analysis of HRs from Cox regression analysis, we found that age, T2DM status and the CCI were inversely associated, and education level positively associated to favorable‐risk prostate cancer (Supporting Information Table 2*a*). Moreover, age was positively associated with aggressive prostate cancer and the CCI inversely. Age, T2DM status use and the CCI were positively associated with death of cardiovascular diseases and other causes, while education levels were inversely associated. Results based on the sub distribution HRs from Fine and Gray regression were similar to the results based on Cox regression (Supporting Information Table 2*b*).

## Discussion

By applying the recent latent class analysis method for competing risks,^31^ we found heterogeneity in risk of prostate cancer with two distinct latent classes in the study population. The risk of prostate cancer, both favorable‐risk and aggressive, was stronger associated with latent class membership than any of the included covariates (age, T2DM status, education level and the CCI). The association with class membership included factors not accounted for in the study, that is class‐specific relative frailty. Moreover, we found no association for T2DM status and prostate cancer risk after isolating the effect of censoring due to competing risks, in contrast to the findings in a conventional survival analysis.

The main strength of the recent method is the possibility to systematically map cohort heterogeneity and the effect of censoring due to competing risks, without violating underlying statistical assumptions. The only assumption made is that any competing risk censoring effects are a consequence of such heterogeneity. Moreover, the method is developed to assess results with straightforward etiological interpretations, in contrast to sub distribution HRs based on competing risk regression.^5^, ^6^ By use of this new method it is possible to detect frail sub classes in the study population with class‐specific risk factors to the event of interest, and to detect scenarios where competing risk may mask or bias etiological associations, such as false aetiology or false protectivity.^9^ A limitation is that information in the covariates can only give an indication about class membership retrospectively. Another limitation is the crude definition of T2DM status based on data of anti‐diabetic drug use without accounting for duration. In the current study, men with T2DM treated with diet only is not included, and a minority of those classified as T2DM might have Type 1 diabetes mellitus, however, this definition of T2DM have been used before in a similar setting.^40^ The results from our study are consistent with studies of frailty of cancers at other sites, where researchers have suggested that a high proportion of cases may arise in a minor susceptible part of the population.^9^, ^41^, ^42^, ^43^ There is one previous study of prostate cancer using the recently developed method where, in line with our results, the population in that study also included two distinct latent classes; however, the frailer class consisted of 16% of the cohort as compared to 9% in our study. 31, 44 The difference in size for the frailer class might be attributed to differences in the overall cohort, as the study by Grundmark *et al*. included a younger cohort with longer follow up, which may imply a higher risk of developing prostate cancer.^44^ We speculate that men in the frail class may have family history of prostate cancer,^45^, ^46^ or an unknown risk factor.

Our conventional analysis of T2DM status and prostate cancer risk showed an inverse association, in line with previous reports.^18^, ^19^, ^20^ The conventional analysis handles all events other than prostate cancer as censored, and neglects the associations with T2DM status and competing risks. To the best of our knowledge, no previous studies have been able to assess the etiological risk of T2DM and risk of prostate cancer, isolated from the effect of censoring from competing risks. By use of the new method we identified the effect of censoring due to competing risks and assessed etiological association isolated from this effect. This resulted in a null association for T2DM status with risk of prostate cancer. We thus noted that the conventional analysis showed what has previously been denoted as false protectivity.^9^, ^31^ There are other reports discussing issues with etiological association after accounting for the effect of censoring from competing risks, these studies have used the terms selection bias of survivors, or competing risk bias.^2^, ^3^, ^4^ Similar to our data, one of these studies investigated the consistent inverse association for smoking and the risk of malignant melanoma, and after simulating and removing the effect of censoring from competing risks, a null etiological association was detected.^2^

In conclusion, methods in conventional survival analysis are often limited by assumptions that are rarely satisfied in real life situations. In the current study we have applied a recently developed method created for heterogeneous populations to calculate etiological risk estimates for prostate cancer undisturbed of competing risks. In accordance with studies of other cancer sites, we found that only a minor part of the studied population had a higher risk of prostate cancer. In line with the concept of precision medicine, this method can detect classes of individuals that differ in their susceptibility to a particular disease, their reaction to a certain risk factor or their response to a specific treatment. These individuals can be further investigated to find clues for preventive or therapeutic interventions.^10^

## Declarations of Interest

None

**Abbreviations**
ALPACA: Advanced Latent Class Prediction And Competing Risk Analysis
CCI: Charlson Comorbidity Index
CI: confidence interval
HR: hazard ratio
PCBaSe: Prostate Cancer Data base Sweden
PSA: prostate‐specific antigen
T2DM: Type 2 diabetes mellitus

## Supporting information

Additional Supporting Information may be found online in the supporting information tab for this article.

Supporting Information

## References

[1] Aalen OO . Heterogeneity in survival analysis. Stat Med. 1988;7:1121–37. 3201038 10.1002/sim.4780071105

[2] Thompson CA , Zhang ZF , Arah OA . Competing risk bias to explain the inverse relationship between smoking and malignant melanoma. Eur J Epidemiol. 2013;28:557–67. 23700026 10.1007/s10654-013-9812-0 PMC3864891

[3] Chang C‐CH , Zhao Y , Lee C‐W , et al. Smoking, death, and Alzheimer's disease: A case of competing risks. Alzheimer Dis Assoc Disord. 2012;26:300 22185783 10.1097/WAD.0b013e3182420b6e PMC3321062

[4] Hernan MA , Alonso A , Logroscino G . Cigarette smoking and dementia: Potential selection bias in the elderly. Epidemiology. 2008;19:448–50. 18414087 10.1097/EDE.0b013e31816bbe14

[5] Andersen PK , Keiding N . Interpretability and importance of functionals in competing risks and multistate models. Stat Med. 2012;31:1074–88. 22081496 10.1002/sim.4385

[6] Austin PC , Fine JP . Practical recommendations for reporting Fine‐Gray model analyses for competing risk data. [Epub ahead of print Sep 15. Stat Med. 2017; (doi: 10.1002/sim.7501.) PMC5698744 28913837

[7] Enroth S , Johansson A , Enroth SB , et al. Strong effects of genetic and lifestyle factors on biomarker variation and use of personalized cutoffs. Nat Commun. 2014;5:4684 25147954 10.1038/ncomms5684 PMC4143927

[8] Zeevi D , Korem T , Zmora N , et al. Personalized Nutrition by Prediction of Glycemic Responses. Cell. 2015;163:1079–94. 26590418 10.1016/j.cell.2015.11.001

[9] Aalen OO , Valberg M , Grotmol T , et al. Understanding variation in disease risk: The elusive concept of frailty. Int J Epidemiol. 2015;44:1408–21. 25501685 10.1093/ije/dyu192 PMC4588855

[10] National Research Council . Toward precision medicine: Building a knowledge network for biomedical research and a new taxonomy of disease. Washington DC: National Academies Press; 2011. 22536618

[11] Fall K , Garmo H , Gudbjornsdottir S , et al. Diabetes mellitus and prostate cancer risk; A nationwide case‐control study within PCBaSe Sweden. Cancer Epidemiol Biomarkers Prev. 2013;22:1102–9. 23580698 10.1158/1055-9965.EPI-12-1046

[12] Bonovas S , Filioussi K , Tsantes A . Diabetes mellitus and risk of prostate cancer: A meta‐analysis. Diabetologia. 2004;47:1071–8. 15164171 10.1007/s00125-004-1415-6

[13] Kasper J , Giovannucci E . A meta‐analysis of diabetes mellitus and the risk of prostate cancer. Cancer Epidemiol Biomarkers Prev. 2006;15:2056 17119028 10.1158/1055-9965.EPI-06-0410

[14] Atchison EA , Gridley G , Carreon JD , et al. Risk of cancer in a large cohort of U.S. veterans with diabetes. Int J Cancer. 2011;128:635–43. 20473855 10.1002/ijc.25362 PMC2962873

[15] Walker JJ , Brewster DH , Colhoun HM , et al. Type 2 diabetes, socioeconomic status and risk of cancer in Scotland 2001‐2007. Diabetologia. 2013;56:1712–5. 23661106 10.1007/s00125-013-2937-6 PMC4131139

[16] Tsilidis KK , Kasimis JC , Lopez DS , et al. Type 2 diabetes and cancer: Umbrella review of meta‐analyses of observational studies. BMJ. 2015;350:g7607 25555821 10.1136/bmj.g7607

[17] Haggstrom C , Van Hemelrijck M , Zethelius B , et al. Prospective study of Type 2 Diabetes Mellitus, anti‐diabetic drugs, and risk of prostate cancer. Int J Cancer. 2016;140:611–7. 27770555 10.1002/ijc.30480 PMC5215657

[18] Preston MA , Riis AH , Ehrenstein V , et al. Metformin use and prostate cancer risk. Eur Urol. 2014;66:1012–20. 24857538 10.1016/j.eururo.2014.04.027

[19] Tseng CH . Metformin significantly reduces incident prostate cancer risk in Taiwanese men with type 2 diabetes mellitus. Eur J Cancer. 2014;50:2831–7. 25201464 10.1016/j.ejca.2014.08.007

[20] Wright JL , Stanford JL . Metformin use and prostate cancer in Caucasian men: Results from a population‐based case‐control study. Cancer Causes Control. 2009;20:1617–22. 19653109 10.1007/s10552-009-9407-y PMC2767519

[21] Discacciati A , Orsini N , Wolk A . Body mass index and incidence of localized and advanced prostate cancer–a dose‐response meta‐analysis of prospective studies. Ann Oncol. 2012;23:1665–71. 22228452 10.1093/annonc/mdr603

[22] Stocks T , Hergens MP , Englund A , et al. Blood pressure, body size and prostate cancer risk in the Swedish Construction Workers cohort. Int J Cancer. 2010;127:1660–8. 20087861 10.1002/ijc.25171

[23] Moller H , Roswall N , Van Hemelrijck M , et al. Prostate cancer incidence, clinical stage and survival in relation to obesity: A prospective cohort study in Denmark. Int J Cancer. 2015;136:1940–7. 25264293 10.1002/ijc.29238

[24] Haggstrom C , Stocks T , Ulmert D , et al. Prospective study on metabolic factors and risk of prostate cancer. Cancer. 2012;118:6199–206. 23090855 10.1002/cncr.27677

[25] Berry SD , Ngo L , Samelson EJ , et al. Competing risk of death: An important consideration in studies of older adults. J Am Geriatr Soc. 2010;58:783–7. 20345862 10.1111/j.1532-5415.2010.02767.x PMC2873048

[26] de Glas NA , Kiderlen M , Vandenbroucke JP , et al. Performing survival analyses in the presence of competing risks: A clinical example in older breast cancer patients. JNCIJ. 2016;108:djv366. 10.1093/jnci/djv366 26614095

[27] Carstensen B , Jorgensen ME , Friis S . The epidemiology of diabetes and cancer. Curr Diab Rep. 2014;14:535 25156543 10.1007/s11892-014-0535-8

[28] Haggstrom C , Stocks T , Nagel G , et al. Prostate cancer, prostate cancer death, and death from other causes, among men with metabolic aberrations. Epidemiology. 2014;25:823–8. 25207955 10.1097/EDE.0000000000000174 PMC4222792

[29] Grundmark B , Garmo H , Loda M , et al. The metabolic syndrome and the risk of prostate cancer under competing risks of death from other causes. Cancer Epidemiol Biomarkers Prev. 2010;19:2088–96. 20647401 10.1158/1055-9965.EPI-10-0112 PMC2923431

[30] Van Hemelrijck M , Garmo H , Holmberg L , et al. Prostate cancer risk in the Swedish AMORIS study: The interplay among triglycerides, total cholesterol, and glucose. Cancer. 2011;117:2086–95. 21523720 10.1002/cncr.25758

[31] Rowley M , Garmo H , Van Hemelrijck M , et al. A latent class model for competing risks. Stat Med. 2017;36:2100–19. 28233395 10.1002/sim.7246

[32] Van Hemelrijck M , Wigertz A , Sandin F , et al. Cohort profile: The National Prostate Cancer Register of Sweden and Prostate Cancer data Base Sweden 2.0. Int J Epidemiol. 2013; 42:956–67. 22561842 10.1093/ije/dys068

[33] Charlson ME , Pompei P , Ales KL , et al. A new method of classifying prognostic co‐morbidity in longitudinal‐studies – Development and validation. J Chronic Dis. 1987;40:373–83. 3558716 10.1016/0021-9681(87)90171-8

[34] Berglund A , Garmo H , Tishelman C , et al. Comorbidity, treatment and mortality: A population based cohort study of prostate cancer in PCBaSe Sweden. J Urol. 2011;185:833–9. 21239002 10.1016/j.juro.2010.10.061

[35] Statistics Sweden . Longitudinal Integration Database for Health Insurance and Labor Market Studies. https://www.scb.se/en_/Services/Guidance‐for‐researchers‐and‐universities/SCB‐Data/Longitudinal‐integration‐database‐for‐health‐insurance‐and‐labour‐market‐studies‐LISA‐by‐Swedish‐acronym/. Accessed October 24, 2017

[36] Ekstrom N , Miftaraj M , Svensson AM , et al. Glucose‐lowering treatment and clinical results in 163 121 patients with type 2 diabetes: An observational study from the Swedish national diabetes register. Diabetes Obes Metab. 2012;14:717–26. 22364580 10.1111/j.1463-1326.2012.01591.x

[37] National Comprehensive Cancer Network . Guidelines 2012. http://www.nccn.org/professionals/physician_gls/pdf/prostate.pdf. Accessed October 24, 2017.

[38] Loeb S , Folkvaljon Y , Damber JE , et al. Testosterone replacement therapy and risk of favorable and aggressive prostate cancer. JCO. 2017;35:1430–6. 10.1200/JCO.2016.69.5304 PMC5455459 28447913

[39] Fine J , Gray R . A proportional hazards model for the subdistribution of a competing risk. J Am Stat Assoc. 1999;94:496–7.

[40] Crawley D , Garmo H , Rudman S , et al. Association between type 2 diabetes, curative treatment and survival in men with intermediate‐ and high‐risk localized prostate cancer. Bju Int. 2018;121:209–16. 28418195 10.1111/bju.13880

[41] Peto J , Mack TM . High constant incidence in twins and other relatives of women with breast cancer. Nat Genet. 2000;26:411–4. 11101836 10.1038/82533

[42] Aalen OO , Tretli S . Analyzing incidence of testis cancer by means of a frailty model. Cancer Causes Control. 1999;10:285–92. 10482487 10.1023/a:1008916718152

[43] Valberg M , Grotmol T , Tretli S , et al. Prostate‐specific antigen testing for prostate cancer: Depleting a limited pool of susceptible individuals? Eur J Epidemiol. 2017; 32:511–20. 27431530 10.1007/s10654-016-0185-z PMC5468491

[44] Grundmark B , Zethelius B , Garmo H , et al. Serum levels of selenium and smoking habits at age 50 influence long term prostate cancer risk; a 34 year ULSAM follow‐up. BMC Cancer. 2011;11:431 21982398 10.1186/1471-2407-11-431 PMC3199281

[45] Bratt O , Drevin L , Akre O , et al. Family history and probability of prostate cancer, differentiated by risk category: A nationwide population‐based study. Jnci J Natl Cancer Inst. 2016;108:djw110. 27400876 10.1093/jnci/djw110

[46] Bratt O , Garmo H , Adolfsson J , et al. Effects of prostate‐specific antigen testing on familial prostate cancer risk estimates. J Natl Cancer Inst. 2010;102:1336–43. 20724726 10.1093/jnci/djq265

