# Supplementary material for: Heterogeneity in risk of prostate cancer: A Swedish population‐based cohort study of competing risks and Type 2 diabetes mellitus
Source: Int J Cancer. 2018 Aug 10;143(8):1868–75. doi: 10.1002/ijc.31587 (PMC6220128; doi:10.1002/ijc.31587)
Supplement: Supplementary file 1 — Supporting Information [file IJC-143-1868-s001.docx]

**Supporting Information Table 1**. Baseline characteristics of the two latent classes found in the study.

|  |  | **Class 1**  **(91%, N=115,623)** | | **Class 2**  **(9%, N=10,859)** | |
| --- | --- | --- | --- | --- | --- |
|  |  | **N** | **%** | **N** | **%** |
|  |  |  |  |  |  |
| **Age at start of study** | 55 to 59 years | 11,946 | 10 | 565 | 5 |
|  | 60 to 64 years | 24,481 | 21 | 1614 | 15 |
|  | 65 to 69 years | 27,593 | 24 | 2181 | 20 |
|  | 70 to 74 years | 22,564 | 20 | 1886 | 17 |
|  | 75 to 79 years | 17,724 | 15 | 1929 | 18 |
|  | 80 to 84 years | 11,315 | 10 | 2684 | 25 |
|  |  |  |  |  |  |
| **Year of selection into comparison cohort** | 2007 | 36,020 | 31 | 3937 | 36 |
|  | 2008 | 35,934 | 31 | 3421 | 32 |
|  | 2009 | 43,669 | 38 | 3501 | 32 |
|  |  |  |  |  |  |
| **Educational level^a^** | Low ^b^ | 47,663 | 41 | 4833 | 45 |
|  | Intermediate | 43,210 | 37 | 3937 | 36 |
|  | High | 24,750 | 21 | 2089 | 19 |
|  |  |  |  |  |  |
| **Charlson Comorbity Index** | No comorbidity (0) | 87,152 | 75 | 6519 | 60 |
|  | Mild comorbidity (1) | 15,228 | 13 | 1606 | 15 |
|  | Moderate comorbidity (2) | 7995 | 7 | 1318 | 12 |
|  | Severe comorbidity (3 or more) | 5248 | 5 | 1416 | 13 |
|  |  |  |  |  |  |
| **Type 2 diabetes mellitus status** | No anti-diabetic drugs | 102,437 | 89 | 9463 | 87 |
|  | Metformin | 4201 | 4 | 351 | 3 |
|  | Insulin/sulphonylurea^c^ | 8985 | 8 | 1045 | 10 |
|  |  |  |  |  |  |

^a^ Educational level categorized as low (≤9 years of school), intermediate (10-12 years), and high (≥13 years), corresponding to mandatory school, high school, and college or university.

^b^ Educational level missing for 2006 men (2%); these men were included in the group with low educational level.

^c^ Ordered variable such as men in the insulin/sulphonylurea group could also use metformin

**Supporting Information Table 2A.** Hazard ratios (HR) calculated by Cox proportional hazards models. HRs are calculated separately for each endpoint, all other endpoints are censored. Each model includes all covariates transformed to z-scores (mean=0, SD=1), and HRs are calculated per unit increase.

|  | **Endpoint** |  |  |  |
| --- | --- | --- | --- | --- |
| **Covariate** | **Diagnosis of favorable-risk prostate cancer** | **Diagnosis of aggressive prostate cancer** | **Death of cardiovascular diseases** | **Death of other causes** |
| **Age** | 0.89 (0.85-0.92) | 1.63 (1.56-1.70) | 2.38 (2.32-2.44) | 1.94 (1.90-1.98) |
| **Type 2 diabetes mellitus status ^a^** | 0.92 (0.88-0.96) | - | 1.07 (1.05-1.08) | 1.05 (1.03-1.07) |
| **Education level^b^** | 1.11 (1.08-1.15) | - | 0.85 (0.83-0.87) | 0.89 (0.87-0.91) |
| **Charlson Comorbidity Index^c^** | 0.91 (0.87-0.95) | 0.93 (0.89-0.98) | 1.54 (1.52-1.57) | 1.46 (1.44-1.48) |

^a^ Defined as 0=no anti-diabetic drugs, 1=metformin, 2=insulin or sulphonylurea

^b^ Defined as 1=low, 2=intermediate, 3=high educational level

^c^ Defined as 0=no comorbidities, 1, 2, 3 or more comorbidities

**Supporting Information Table 2B**. Sub distribution hazard ratios (HR) calculated by Fine and Gray regression models. SHRs are calculated separately for each endpoint, all other endpoints handled as competing events. Each model includes all covariates transformed to z-scores (mean=0, SD=1), and HRs are calculated per unit increase.

|  | **Endpoint** |  |  |  |
| --- | --- | --- | --- | --- |
| **Covariate** | **Diagnosis of favorable-risk prostate cancer** | **Diagnosis of aggressive prostate cancer** | **Death of cardiovascular diseases** | **Death of other causes** |
| **Age** | 0.84 (0.81-0.86) | 1.52 (1.46-1.58) | 2.22 (2.16-2.28) | 1.82 (1.78-1.85) |
| **Type 2 diabetes mellitus status ^a^** | 0.91 (0.88-0.95) | - | 1.06 (1.04-1.08) | 1.04 (1.03-1.06) |
| **Education level^b^** | 1.12 (1.09-1.16) | - | 0.86 (0.84-0.88) | 0.90 (0.88-0.92) |
| **Charlson Comorbidity Index^c^** | 0.85 (0.82-0.89) | 0.86 (0.83-0.90) | 1.45 (1.43-1.47) | 1.38 (1.36-1.40) |

^a^ Defined as 0=no anti-diabetic drugs, 1=metformin, 2=insulin or sulphonylurea

^b^ Defined as 1=low, 2=intermediate, 3=high educational level

^c^ Defined as 0=no comorbidities, 1, 2, 3 or more comorbidities
